# Supplementary material for: Real-space heterogeneous reconstruction, refinement, and disentanglement of CryoEM conformational states with HetSIREN
Source: bioRxiv. 2024 Sep 19:2024.09.16.613176. Preprint. [Version 1] doi: 10.1101/2024.09.16.613176 (PMC11429808; doi:10.1101/2024.09.16.613176)
Supplement: 1 [file NIHPP2024.09.16.613176v1-supplement-1.pdf]

bioRxiv preprint doi: https://doi.org/10.1101/2024.09.16.613176; this version posted September 19, 2024. The copyright holder for this preprint (which was not certified by peer review) is the author/funder, who has granted bioRxiv a license to display the preprint in perpetuity. It is made available under aCC-BY 4.0 International license.

Supplementary Methods

Cryo-EM sample preparation and data collection for the SARS-CoV-2 samples

0.5 mg/ml of purified Beta variant Spike protein sample in 1X PBS buffer at pH 7 was diluted by 100 mM sodium citrate tribasic dihydrate at pH 5 to a final concentration of 0.25 mg/ml and a final pH value of 5.5, a condition in which the preferred orientation was minimized. A 4 μl sample was applied to Quantifoil holey carbon grids R1.2/1.3 200 mesh for 4°C, and to Quantifoil gold grids R2/2 200 mesh for 37°C, with prior incubation at the respective temperatures for 10 min. The grids had been glow discharged with negative polarity at 25 mA for 30 seconds using an EMS 100 Glow discharge apparatus. They were used within 30 min to minimize the hydrophilic recovery of the grids. After application of the sample, the grids were incubated for 10 seconds in 100% humidity at 4°C or 37°C in a Mark IV vitrobot device (Thermo Fisher Scientific) and then blotted for 3 seconds with blot force 0 before being flash frozen in liquid ethane cooled by liquid nitrogen.

For the 4°C and 37°C samples, 11,137 and 7,064 movie micrographs were automatically collected on a Bio-quantum-K3 detector (Gatan, Inc.) at a nominal magnification of 81,000x which resulted in a pixel size of 1,061 Å by using a Titan Krios microscope (Thermo Fisher Scientific) operating at 300 keV with

bioRxiv preprint doi: https://doi.org/10.1101/2024.09.16.613176; this version posted September 19, 2024. The copyright holder for this preprint (which was not certified by peer review) is the author/funder, who has granted bioRxiv a license to display the preprint in perpetuity. It is made available under aCC-BY 4.0 International license.

a GIF Quantum energy filter with a slit width of 20 eV. 50 frames per movie were collected at 1 e-/ Å^2 per frame for a total dose of 50 e-/ Å^2 on the sample by using counting mode at a defocus range between -1.5 μm to -2.2 μm.

Standard image processing workflow for the SARS-CoV-2 samples

All image processing steps were performed within the Scipion software framework (17). For both samples, particles were previously pooled through standard 2D classification approaches in CryoSPARC (36) conducted by the laboratory of Prof. Ming-Daw Tsai. These particles were then directly imported into Scipion, with 662,379 and 468,911 particles for the samples at 4°C and 37°C, respectively. The selected particles were downsampled to 1.4 Å/px. These particles generated four *ab initio* models imposing C3 symmetry in CryoSPARC (36). All particles were subjected to non-uniform refinement using the best initial model low-pass filtered to 30 Å as a reference. This refinement was followed by an angular consensus protocol (37), retaining the best 615,000 and 410,000 particles for the samples at 4°C and 37°C, respectively. We then symmetry-relaxed these C3 symmetry-refined particles into C1 (38) while performing a 3D classification into 10 classes as implemented in Relion (39). We employed a 3D clustering consensus protocol to retain stable and statistically significant particles across the entire datasets to minimize the variability in class distribution over replicates of the same protocol. We inspected the particle clusters with a p-value $<$ 0.05, and to confirm the assignment of particles to the different conformations, we generated initial models and non-uniform refined them independently. We then rejected the clusters of particles resulting in junk volumes and selected only the best clusters, corresponding to 479,908 and 309,062 particles for the samples at 4°C and 37°C, respectively. After this standard image processing workflow, we merged all clusters for each independent sample and subjected the corresponding particles to HetSIREN.

Model building and refinement of the SARS-CoV-2 samples

Firstly, we manually docked the model into the density as a rigid body, followed by real space fitting using the Fit in Map routine in UCSF Chimera (40) for the complete Spike structure, which includes chains A, B, and C. We used previously deposited structures as starting models, matching each detected conformation: PDB IDs 7WEV and 7VX1 for Beta variant in the 3 Down and 1 Up states, respectively (41). For the 2 Up state, we computationally modified the 1 Up model (7VX1) by removing one of the RBD Down chains and replacing it with a previously duplicated and individually fitted RBD Up chain. Real-space refinement was then performed in Phenix (**?**) with the enabled global minimization, local grid search, ADP, and rigid body options. We defined each chain's NTD, RBD, and S2 domains as independent rigid bodies, resulting in 9 rigid bodies in total. To preserve the general arrangement of the different domains within the Spike protein, the starting model was used as a reference model with restraints

bioRxiv preprint doi: https://doi.org/10.1101/2024.09.16.613176; this version posted September 19, 2024. The copyright holder for this preprint (which was not certified by peer review) is the author/funder, who has granted bioRxiv a license to display the preprint in perpetuity. It is made available under aCC-BY 4.0 International license.

and secondary structure restraints. The resulting models were then manually inspected in Chimera (40) and Coot (42) to check the fit to the density. The quality of the obtained models was assessed using MolProbity (43) as implemented in Phenix (44) and the Worldwide PDB (wwPDB) OneDep System (https://deposit-pdbe.wwpdb.org/deposition). Refinement statistics are listed in Supplementary Table 2 and 3.

bioRxiv preprint doi: https://doi.org/10.1101/2024.09.16.613176; this version posted September 19, 2024. The copyright holder for this preprint (which was not certified by peer review) is the author/funder, who has granted bioRxiv a license to display the preprint in perpetuity. It is made available under aCC-BY 4.0 International license.

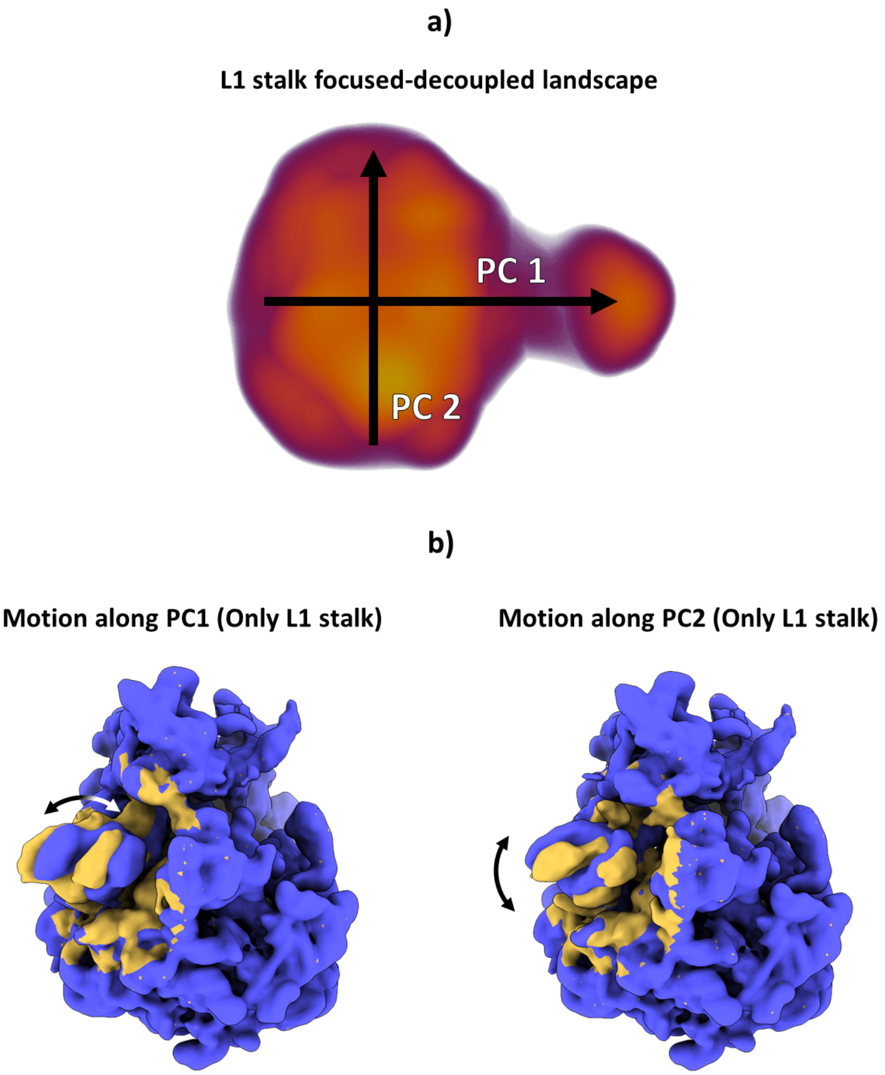

Supplementary Figure 1: Example of the L1 stalked-focused landscape estimated with HetSIREN. The landscape was estimated with the pose and CTF decoupling architecture by providing a spherical mask to the network enclosing the L1 stalk. This way, HetSIREN will only consider the L1 stalk region when determining the motions and conformational changes captured in the experimental particle images. Panel a) shows the UMAP representation of the conformational latent space, including the approximate principal direction according to PCA. Panel b) shows the main L1 stalk motions detected by HetSIREN when sampling along the conformational latent space's first and second principal components. The motion detected shows a strong lateral and vertical displacement of the L1 stalk, which is much more easily identified here than when considering the whole particle, thanks to the ability to focus the landscape in this specific region.

bioRxiv preprint doi: https://doi.org/10.1101/2024.09.16.613176; this version posted September 19, 2024. The copyright holder for this preprint (which was not certified by peer review) is the author/funder, who has granted bioRxiv a license to display the preprint in perpetuity. It is made available under aCC-BY 4.0 International license.

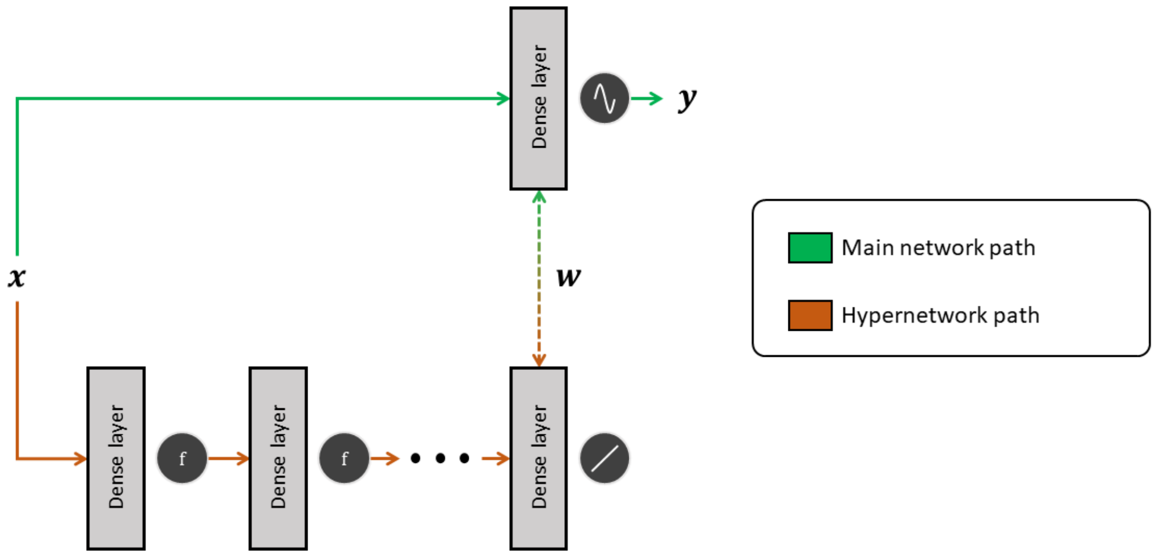

Supplementary Figure 2: Scheme of a meta-sinusoidal layer as implemented in the HetSIREN volume decoder network. The proposed architecture relies on a fully connected network with several layers (hypernetwork) whose weights will be updated during the backpropagation phase. The weights of the last layer in the fully connected network are then shared with the dense layer with the sine activation so that it can decode the appropriate outputs.

bioRxiv preprint doi: https://doi.org/10.1101/2024.09.16.613176; this version posted September 19, 2024. The copyright holder for this preprint (which was not certified by peer review) is the author/funder, who has granted bioRxiv a license to display the preprint in perpetuity. It is made available under aCC-BY 4.0 International license.

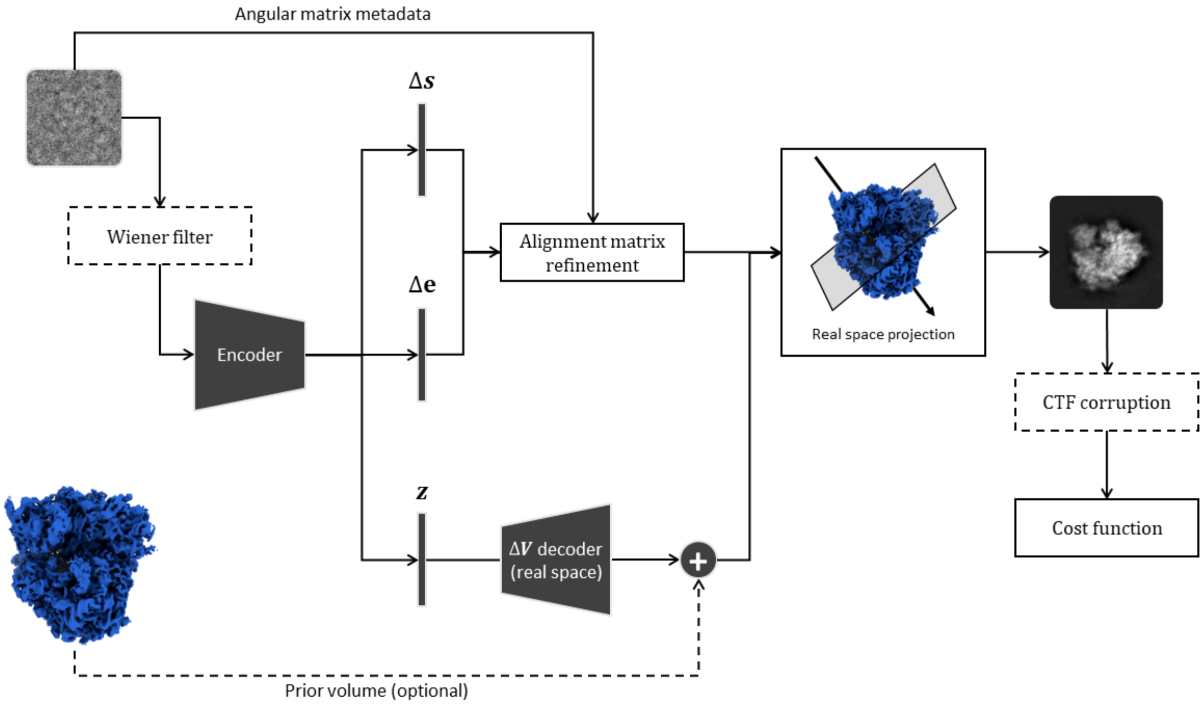

Supplementary Figure 3: Scheme of the HetSIREN network architecture and training strategy. In the scheme, the encoder has a dynamic architecture based on the user inputs (available choices include fully connected and convolutional architectures). The $\Delta\mathbf{V}$ decoder directly produces a full 3D volume in real space from the encoded latent space vectors $\mathbf{z}$. Depending on the availability of the prior volume, the decoded $\Delta\mathbf{V}$ could translate into a full reconstruction (without the prior volume) or a refinement. In addition to the conformational latent space $\mathbf{z}$, two additional bottleneck layers are estimated: a $\Delta\boldsymbol{s}$ layer to refine the in-plane shift of the particle and a $\Delta\boldsymbol{e}$ layer to refine the particle projection angle. The previous two vectors are combined to refine the estimated alignment matrices associated with the experimental image. Regarding the CTF, three possible scenarios are considered: particles have been previously corrected (no CTF considered inside the network), particles are CTF corrected before being fed to the encoder (Wiener filter box), or theoretical projections are CTF corrupted (CTF corruption box).

bioRxiv preprint doi: https://doi.org/10.1101/2024.09.16.613176; this version posted September 19, 2024. The copyright holder for this preprint (which was not certified by peer review) is the author/funder, who has granted bioRxiv a license to display the preprint in perpetuity. It is made available under aCC-BY 4.0 International license.

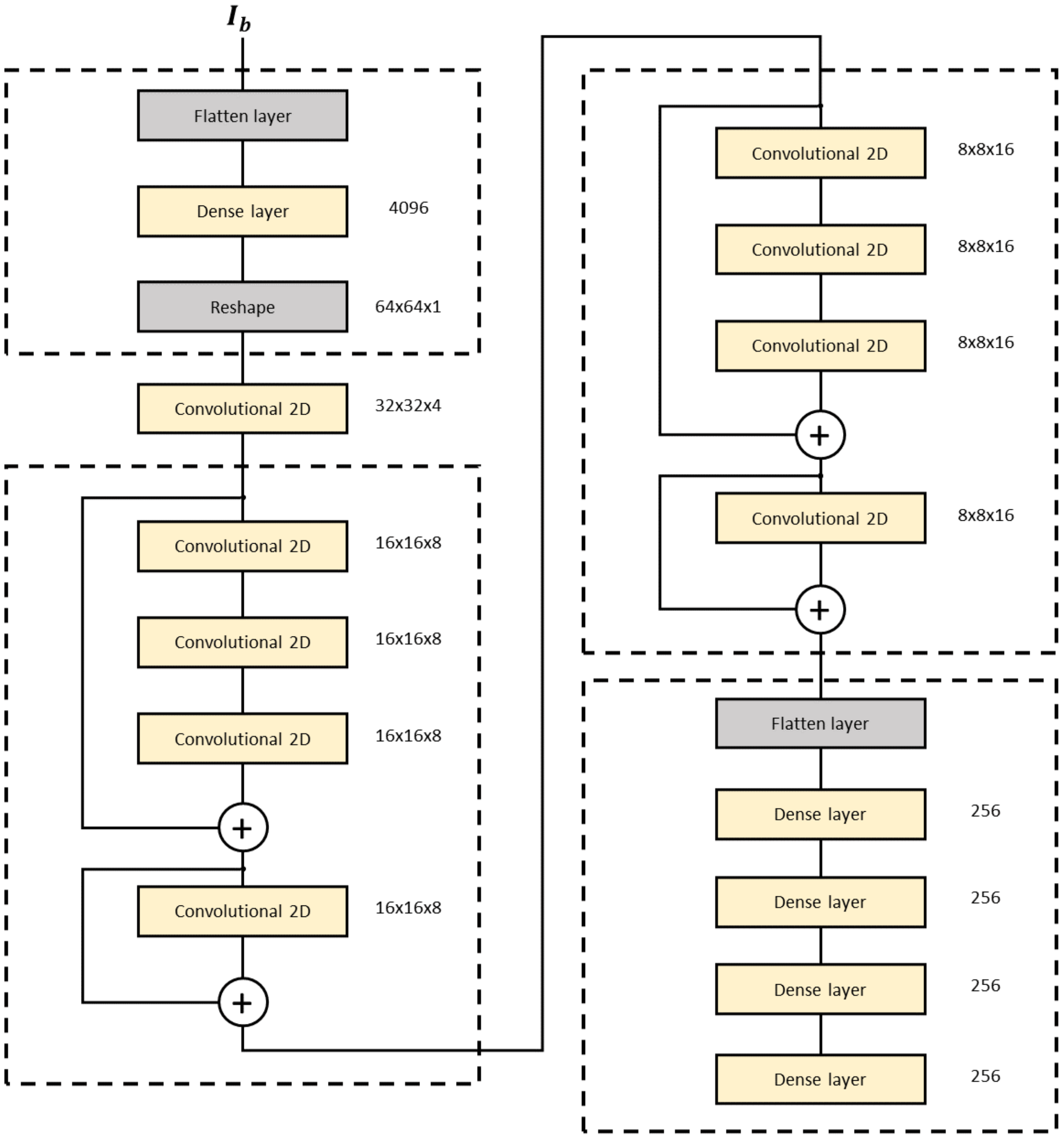

Supplementary Figure 4: Example of the default encoder architecture implemented in HetSIREN. The encoder relies on a resizing network followed by convolutional blocks with residual skips. The output images from the residual blocks are then passed to a fully connected block whose output is posteriorly converted into the three bottlenecks defined in HetSIREN.

bioRxiv preprint doi: https://doi.org/10.1101/2024.09.16.613176; this version posted September 19, 2024. The copyright holder for this preprint (which was not certified by peer review) is the author/funder, who has granted bioRxiv a license to display the preprint in perpetuity. It is made available under aCC-BY 4.0 International license.

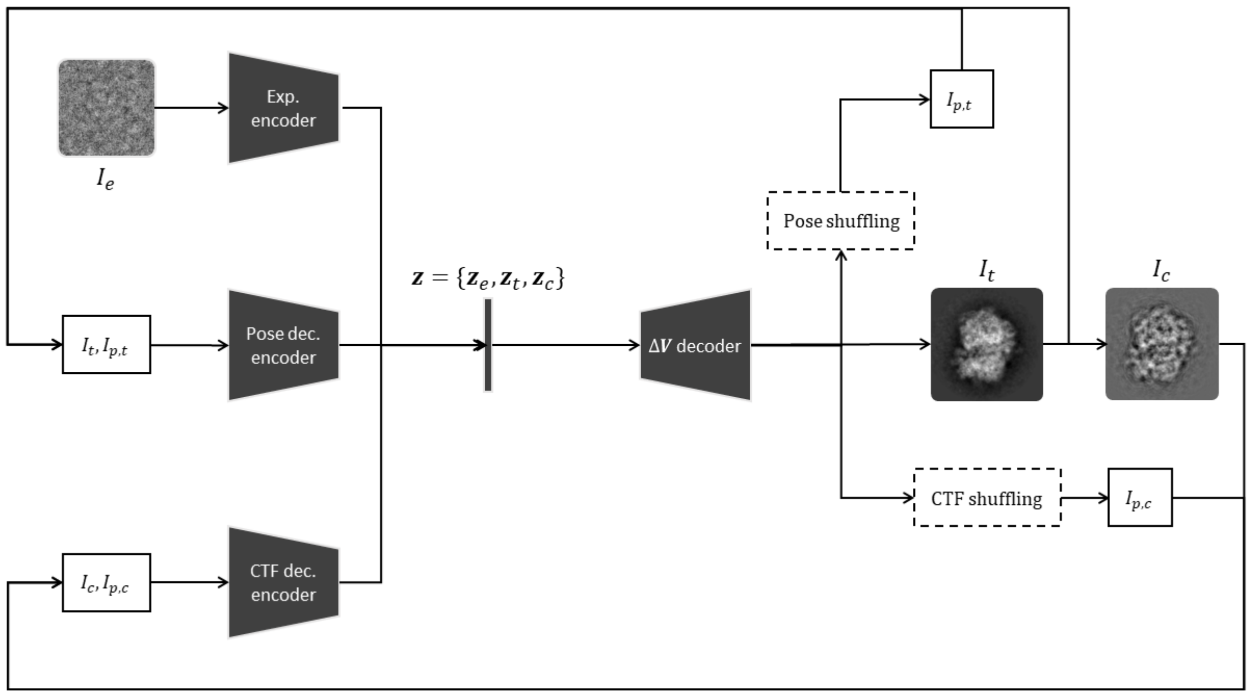

Supplementary Figure 5: HetSIREN poses and CTF decoupling architecture. The decoupling process starts with a batch of experimental images forwarded through the experimental encoder and the decoder to generate a batch of clean, and CTF corrupted projections I_t and I_c. In addition, the original poses and CTFs are shuffled to generate a new set of clean projections with the same conformation but variable pose and CTF $I_{p,t}$ and $I_{p,c}$. Once all the projections have been generated, the images I_t and $I_{p,t}$ are forwarded through the pose decoupling decoder. Similarly, the images I_c and $I_{p,c}$ are fed to the CTF decoupling decoder. In this way, it is possible to generate several sets of latent space vectors representing the same conformational state but with variable pose and CTF, which can be used to decouple the pose and CTF effects from the latent space as expressed in Equations 3 and 4.

bioRxiv preprint doi: https://doi.org/10.1101/2024.09.16.613176; this version posted September 19, 2024. The copyright holder for this preprint (which was not certified by peer review) is the author/funder, who has granted bioRxiv a license to display the preprint in perpetuity. It is made available under aCC-BY 4.0 International license.

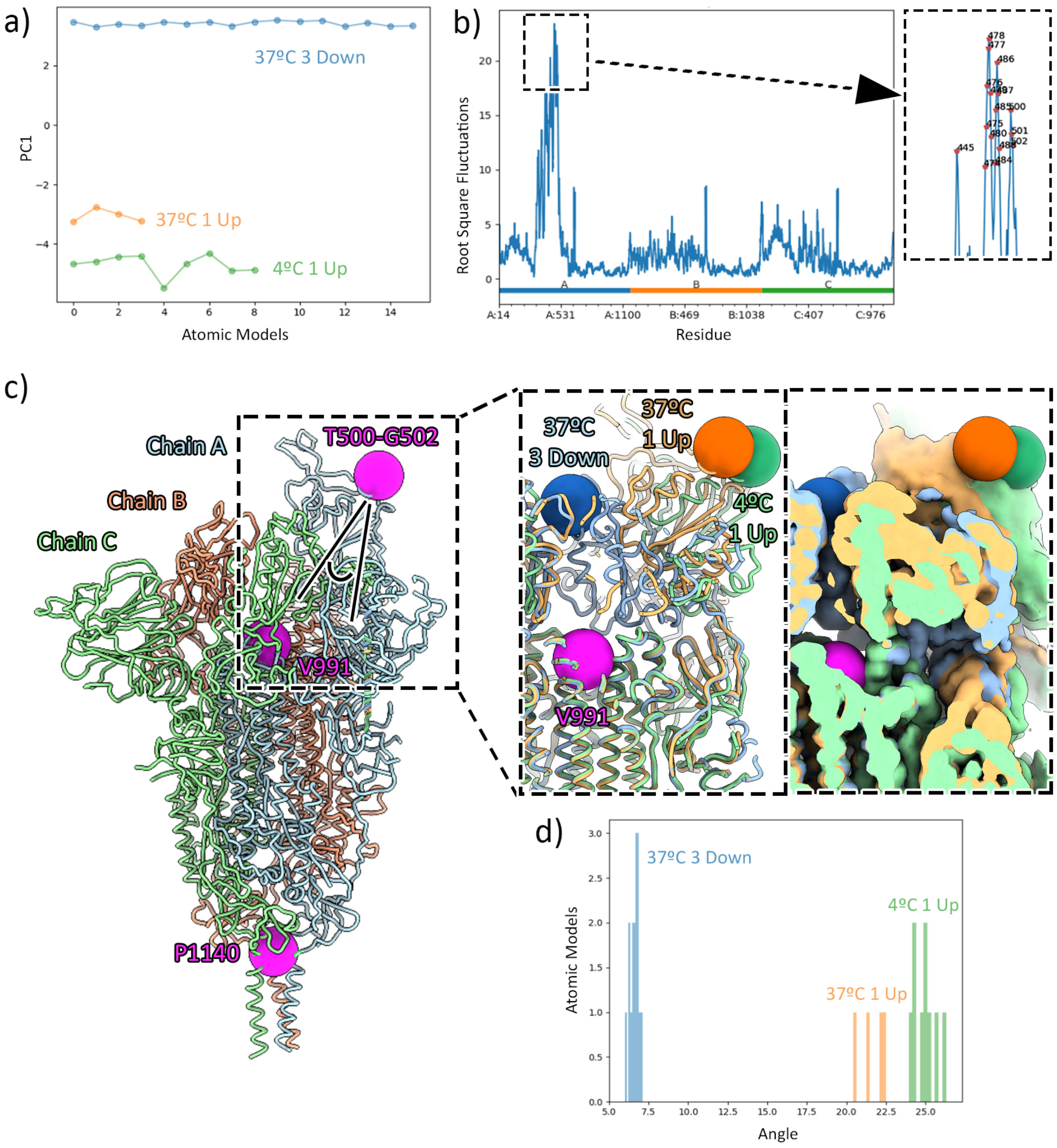

Supplementary Figure 6: Structural analyses of the SARS-CoV-2 Spike protein. Panel a) shows the PCA for the atomic structures ensemble encompassing 16 models of 3 Down conformation at 37°C (blue), 4 models of 1 Up conformation at 37°C (orange), and 9 models of 1 Up conformation at 4°C (green). The Root Mean Square Fluctuations derived from PCA for individual residues are shown in panel b). The inset shows a zoomed area of the residues exhibiting the highest mobility. Panel c) shows a representative atomic model for the 1 Up conformation (chain A in light blue, chain B in light red, and chain C in light green). The three magenta spheres represent the centroids used for the analyses of angle measurements (Thr500-Gly502 at the RBD and Val991 and Pro1140 at the top and bottom of the S2 domain, respectively). Angle is indicated in the dashed box. Insets show a detail of the differences between the three analyzed conformations (3 Down at 37°C in light blue, 1 Up at 37°C in light orange, and 1 Up at 4°C in light green) at atomic models (left) and cryoEM reconstructions (right) levels. Analyses of angle measurements are shown in panel d), matching the color code of inset c).

bioRxiv preprint doi: https://doi.org/10.1101/2024.09.16.613176; this version posted September 19, 2024. The copyright holder for this preprint (which was not certified by peer review) is the author/funder, who has granted bioRxiv a license to display the preprint in perpetuity. It is made available under aCC-BY 4.0 International license.

	RBD (residues 304-591)
**CryoSPARC Map**<br>**Modelled Residues**<br>**% Total Residues**	310<br>35.9%
**HetSIREN map 4**	365<br>42.2%
**HetSIREN map 9**	342<br>39.6%
**HetSIREN map 13**	401<br>46.4%
**HetSIREN map 14**	326<br>37.7%
**HetSIREN map 15**	346<br>40.0%

Supplementary Table 1: Comparison of automatically modeled residues performed by ModelAngelo.

bioRxiv preprint doi: https://doi.org/10.1101/2024.09.16.613176; this version posted September 19, 2024. The copyright holder for this preprint (which was not certified by peer review) is the author/funder, who has granted bioRxiv a license to display the preprint in perpetuity. It is made available under aCC-BY 4.0 International license.

Refinement	Map 1	Map 2	Map 3	Map 4	Map 5	Map 6	Map 7	Map 8	Map 9	Map 10
Mask correlation coefficient	0.69	0.69	0.70	0.71	0.64	0.69	0.70	0.72	0.72	0.69
Model composition										
Non-hydrogen atoms	25,362	25,362	25,362	25,362	25,362	25,362	25,362	25,362	25,362	25,362
Protein residues	3,237	3,237	3,237	3,237	3,237	3,237	3,237	3,237	3,237	3,237
ADP (B-factors)										
min	56.88	65.32	54.68	67.54	47.65	52.98	59.54	50.81	63.09	62.66
max	428.31	325.45	462.43	318.58	363.74	620.24	539.35	297.54	358.72	455.62
mean	149.62	132.17	156.76	138.32	155.21	157.98	161.59	136.61	131.11	156.43
R.m.s deviations										
Bond lengths	0.007	0.007	0.007	0.007	0.007	0.007	0.007	0.007	0.009	0.008
Bond angles	1.428	1.490	1.442	1.500	1.367	1.384	1.446	1.484	1.597	1.455
Validation										
Molprobity score	1.65	1.77	1.62	1.81	1.53	1.60	1.58	1.64	1.79	1.69
Clashscore	5.91	6.35	5.95	6.31	4.97	5.77	5.55	6.49	7.64	6.99
Rotamer outliers (%)	1.17	0.74	1.06	0.99	0.78	0.67	0.88	0.99	1.24	0.60
Ramachandran plot										
Favoured (%)	95.97	93.05	95.94	95.69	96.03	95.91	96.00	95.91	95.72	95.66
Allowed (%)	3.97	6.95	4.00	4.25	3.91	4.03	3.94	4.03	4.22	4.28
Outlier (%)	0.06	0.00	0.06	0.06	0.06	0.06	0.06	0.06	0.06	0.06

Refinement	Map 11	Map 12	Map 13	Map 14	Map 15	Map 16	Map 17	Map 18	Map 19	Map 20
Mask correlation coefficient	0.68	0.70	0.71	0.72	0.70	0.67	0.69	0.69	0.69	0.71
Model composition										
Non-hydrogen atoms	25,362	25,362	25,362	25,362	25,362	25,362	25,362	25,362	25,362	25,362
Protein residues	3,237	3,237	3,237	3,237	3,237	3,237	3,237	3,237	3,237	3,237
ADP (B-factors)										
min	54.78	55.29	55.46	58.05	51.09	54.05	55.09	58.39	49.91	51.77
max	579.72	549.59	440.90	259.33	375.42	470.42	454.68	401.38	460.43	657.55
mean	156.63	171.29	150.96	127.42	140.63	155.53	153.43	144.97	159.05	153.25
R.m.s deviations										
Bond lengths	0.008	0.007	0.008	0.008	0.008	0.007	0.007	0.007	0.007	0.007
Bond angles	1.478	1.441	1.608	1.532	1.473	1.402	1.465	1.430	1.433	1.515
Validation										
Molprobity score	1.72	1.58	1.65	1.67	1.61	1.58	1.61	1.69	1.58	1.66
Clashscore	6.17	5.47	6.31	6.73	5.89	5.63	6.05	6.93	5.29	6.47
Rotamer outliers (%)	1.41	0.78	0.81	0.92	0.99	0.67	0.81	1.09	0.78	0.81
Ramachandran plot										
Favoured (%)	96.03	95.91	95.66	95.66	95.78	95.97	95.97	95.91	95.72	95.69
Allowed (%)	3.91	4.03	4.25	4.28	4.15	3.97	3.97	4.00	4.22	4.25
Outlier (%)	0.06	0.06	0.09	0.06	0.06	0.06	0.06	0.09	0.06	0.06

Supplementary Table 2: Refinement statistics for SARS-CoV-2 Spike protein at 4°C.

bioRxiv preprint doi: https://doi.org/10.1101/2024.09.16.613176; this version posted September 19, 2024. The copyright holder for this preprint (which was not certified by peer review) is the author/funder, who has granted bioRxiv a license to display the preprint in perpetuity. It is made available under aCC-BY 4.0 International license.

**Refinement**	**Map 1**	**Map 2**	**Map 3**	**Map 4**	**Map 5**	**Map 6**	**Map 7**	**Map 8**	**Map 9**	**Map 10**
Mask correlation coefficient	0.68	0.69	0.74	0.77	0.73	0.75	0.76	0.74	0.69	0.73
Model composition										
Non-hydrogen atoms	25,362	25,362	25,482	25,482	25,482	25,482	25,482	25,482	25,362	25,482
Protein residues	3,237	3,237	3,255	3,255	3,255	3,255	3,255	3,255	3,237	3,255
ADP (B-factors)										
min	66.33	69.60	73.86	84.37	69.29	71.30	68.22		73.47	63.30
max	469.35	418.14	418.14	331.04	299.72	277.98	319.64	261.50	412.59	336.72
mean	151.83	149.02	197.15	128.96	134.03	124.63	127.39	124.20	151.80	135.49
R.m.s deviations										
Bond lengths	0.008	0.008	0.008	0.009	0.009	0.008	0.009	0.009	0.007	0.008
Bond angles	1.603	1.612	1.717	1.840	1.829	1.745	1.777	1.797	1.566	1.738
Validation										
Molprobity score	1.69	1.65	1.61	1.80	1.62	1.66	1.59	1.63	1.68	1.63
Clashscore	6.63	6.21	6.06	7.53	6.99	6.26	6.36	6.49	6.69	5.96
Rotamer outliers (%)	1.02	0.88	1.16	1.72	0.88	1.33	1.05	1.19	0.92	1.33
Ramachandran plot										
Favoured (%)	95.44	95.50	96.49	96.71	96.49	96.58	96.52	96.61	95.60	96.71
Allowed (%)	4.47	4.40	3.42	3.20	3.42	3.32	3.39	3.29	4.31	3.20
Outlier (%)	0.09	0.09	0.09	0.09	0.09	0.09	0.09	0.09	0.09	0.09

**Refinement**	**Map 11**	**Map 12**	**Map 13**	**Map 14**	**Map 15**	**Map 16**	**Map 17**	**Map 18**	**Map 19**	**Map 20**
Mask correlation coefficient	0.67	0.74	0.75	0.76	0.74	0.74	0.74	0.74	0.75	0.74
Model composition										
Non-hydrogen atoms	25,362	25,482	25,482	25,482	25,482	25,482	25,482	25,482	25,482	25,482
Protein residues	3,237	3,255	3,255	3,255	3,255	3,255	3,255	3,255	3,255	3,255
ADP (B-factors)										
min	67.35	73.33	75.37	80.29	70.45	66.29	71.37	86.52	76.29	78.77
max	450.55	359.03	350.66	288.60	290.02	330.95	288.01	405.81	421.83	304.79
mean	152.38	130.67	127.95	128.29	127.89	129.61	125.55	131.54	262.12	125.21
R.m.s deviations										
Bond lengths	0.007	0.009	0.008	0.008	0.008	0.008	0.008	0.008	0.008	0.008
Bond angles	1.549	1.793	1.751	1.739	1.740	1.790	1.812	1.720	1.799	1.746
Validation										
Molprobity score	1.70	1.66	1.60	1.58	1.65	1.59	1.69	1.59	1.61	1.57
Clashscore	5.69	6.36	6.57	6.16	6.32	6.16	6.87	5.84	6.45	5.44
Rotamer outliers (%)	0.85	1.37	1.09	1.09	1.30	1.12	1.30	1.19	1.19	1.23
Ramachandran plot										
Favoured (%)	95.56	96.74	96.64	96.68	96.64	96.64	96.55	96.64	96.77	96.71
Allowed (%)	4.34	3.17	3.26	3.23	3.26	3.26	3.29	3.26	3.11	3.20
Outlier (%)	0.09	0.09	0.09	0.09	0.09	0.09	0.16	0.09	0.12	0.09

Supplementary Table 3: Refinement statistics for SARS-CoV-2 Spike protein at 37°C.
